# Rescue Therapies for *H. pylori* Infection in Italy

**DOI:** 10.3390/antibiotics10050525

**Published:** 2021-05-03

**Authors:** Vincenzo De Francesco, Angelo Zullo, Luigi Gatta, Raffaele Manta, Matteo Pavoni, Ilaria Maria Saracino, Giulia Fiorini, Dino Vaira

**Affiliations:** 1 Gastroenterology Unit, ‘Riuniti’ Hospital, 71122 Foggia, Italy; 2 Gastroenterology and Digestive Endoscopy, Nuovo Regina Margherita Hospital, 00153 Rome, Italy; angelomario.zullo@aslroma1.it; 3 Gastroenterology and Endoscopy Unit, Versilia Hospital, 55049 Lido di Camaiore, Italy; gattalg@gmail.com; 4 Gastroenterology and Digestive Endoscopy, ‘Generale’ Hospital, 06129 Perugia, Italy; r.manta@libero.it; 5 Department of Medical and Surgical Sciences, S. Orsola Hospital, University of Bologna, 40138 Bologna, Italy; matteo.pavoni@studio.unibo.it (M.P.); ilariamaria.saracino@studio.unibo.it (I.M.S.); giulia.fiorini@aosp.bo.it (G.F.); berardino.vaira@unibo.it (D.V.)

**Keywords:** *Helicobacter pylori*, rescue therapy, retreatment, antibiotic, therapy regimens

## Abstract

**Background/Aims**: Curing *Helicobacter pylori* infection remains challenging for clinicians, as no proposed first-line therapy achieves bacterial eradication in all treated patients so that several patients need two or more consecutive treatments. Bacterial culture with antibiotics susceptibility testing is largely unachievable in Italy, and empiric second-line and rescue therapies are generally used. This study aimed to identify what eradication regimens perform better in Italy, following first-line therapy failure. **Methods:** We performed a literature search on PubMed for studies on standard therapy regimens used as second-line or rescue treatments performed in adult patients. Studies including modified drug combinations were not considered. Both intention-to-treat and per- protocol analyses were computed for each therapy subgroup. **Results:** Data from 35 studies with a total of 4830 patients were eventually considered. As a second-line therapy, Pylera^®^ (90.6%) and a sequential regimen (89.8%) achieved eradication rates significantly higher than other therapies. For third-line therapy, a levofloxacin-based regimen and Pylera^®^ achieved comparable eradication rates (88.2% vs. 84.7%; *p* = 0.2). Among therapies used as fourth (or more) attempts, Pylera^®^ and a rifabutin-based therapy achieved 77.4% and 66.4% cure rates, respectively (*p* = 0.013). A therapy sequence based on the type of first-line therapy used was proposed. **Conclusions:** Data obtained through our review indicate that standard therapies for *H. pylori* eradication can be used when following an appropriate sequence, allowing clinicians to improve the cure rate without resorting to bacterial culture.

## 1. Introduction

*Helicobacter pylori* still remains a widely diffuse infection worldwide [1], and it is the main cause of both benign and malignant gastroduodenal diseases, including non-ulcer dyspepsia, peptic ulcers, gastric MALT-lymphoma and carcinoma, as well as interaction with non-steroidal anti-inflammatory drugs in causing gastroduodenal lesions [2,3]. Moreover, *H. pylori* plays a definite role in some extra-intestinal disorders, such as idiopathic thrombocytopenic purpura and idiopathic iron deficiency anemia [4,5], whilst the association with other diseases (neurological, dermatological, hematologic, ocular, cardiovascular, metabolic, allergic, and hepatobiliary diseases) deserves further investigation [6]. It is well known that *H. pylori* eradication definitely changes the natural history of peptic ulcer disease, in terms of recurrence and complications. Moreover, curing the infection reduces the risk of precancerous lesion evolution in the stomach. *H. pylori* eradication is not easily achieved, since several bacterial and host factors are involved in the therapeutic success, so that an antibiotic combination is required [7]. In detail, bacterial resistance towards antibiotics in *H. pylori* isolates is increasing [8]. Different therapy schedules, combining the few currently available antibiotics proven to be active against *H. pylori* strains in gastric juices, have been proposed. However, the best first-line therapies currently proposed achieve an eradication rate of around 90%, so that about 10% of patients would need retreatment [9]. When considering the large prevalence of this infection worldwide, such a figure corresponds to millions of patients. Unfortunately, curing the infection following previous therapeutic attempts is definitively more difficult, largely due to secondary bacterial resistance towards the few available antibiotics for the eradication regimens, and the reduction of patients’ compliance [10]. Current Italian and European guidelines suggest that a susceptibility-based therapy should be prescribed following two or more consecutive failures [2,10]. However, this approach is largely impracticable in clinical practice. Indeed, the need for dedicated microbiological laboratories, the challenges in isolating *H. pylori* from gastric biopsies, and the lack of constant correspondence between in vitro and in vivo results in terms of eradication, represent unsurmountable limitations for this approach [11,12]. Therefore, different empiric therapeutic regimens are currently used as rescue therapy, including a proton pump inhibitor (PPI) and levofloxacin-based, rifabutin-based, high-dose dual, sequential, concomitant, hybrid and bismuth-based therapies [13]. However, there is no one therapy sequence clearly proven to be more successful than another, causing uncertainty in choosing rescue therapies. What is the evidence regarding rescue therapies in Italy? To answer this question, we performed a review of the available literature with pooled data analysis.

## 2. Results

### 2.1. Descriptive Analysis

**Figure 1:**
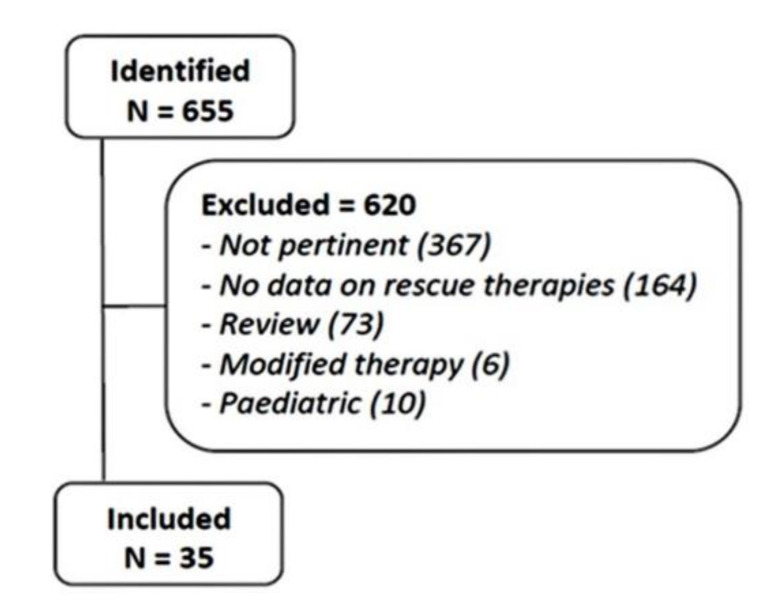
The flow chart of the literature review.

A total of 655 citations were found on PubMed. Following title and abstract reviews, 35 studies met the inclusion criteria, with 72 therapeutic arms and 4830 patients [14,15,16,17,18,19,20,21,22,23,24,25,26,27,28,29,30,31,32,33,34,35,36,37,38,39,40,41,42,43,44,45,46,47,48], whilst the others were ultimately excluded for different reasons, as listed in Figure 1. Overall, there were 26 therapeutic arms on a levofloxacin-based therapy, 25 on a bismuth-based quadruple therapy, including 21 studies with PPI and Pylera^®^ (a standardized three-in-one capsule, containing bismuth subcitrate potassium (140 mg), metronidazole (125 mg), and tetracycline (125 mg), and 4 studies with standard PPI, bismuth, tetracycline, and metronidazole; 16 on rifabutin-based, and 5 on sequential therapy, whilst no study on concomitant, hybrid or high-dose dual therapy was available. Data from the second-line to the fifth attempt were available, and they were accordingly analyzed. The study design of 35 retrieved publications was prospective in 28 publications and retrospective in 7, and the enrollment was multicenter in 23 and single-center in 12.

### 2.2. Eradication Rates

The eradication rates achieved by rescue treatments are reported in Table 1 at “per protocol” (PP) and at “intention-to-treatment” (ITT) analysis. As a second-line therapy, bismuth-based quadruple therapies (Pylera^®^ or classic regimen) and a sequential regimen achieved the highest eradication rates. In detail, the cure rate following bismuth-based quadruple therapies was significantly higher than that of levofloxacin-based (*p* < 0.001) and rifabutin-based (*p* < 0.001) regimens, and similar to that of sequential therapy (*p* = 0.8). As a third-line therapy, a levofloxacin-based regimen and Pylera^®^ achieved comparable eradication rates (88.2% vs. 84.7%; *p* = 0.2), whilst the classic bismuth-based therapy achieved the lowest cure rate. Among the therapies used, three (or more) attempts failed; only rifabutin and Pylera^®^ were tested in a significant number of patients, reporting an eradication rate of 66.4% and 77.4%, respectively (*p* = 0.013). For the other regimens assessed, the number of patients evaluated was too low to be able to draw conclusions.

## 3. Discussion

**Figure 2:**
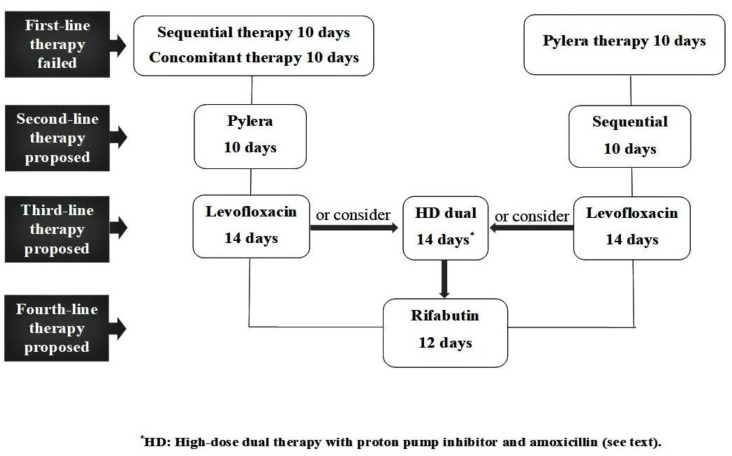
The proposed therapies sequence.

*H. pylori* still remains a widely diffuse infection worldwide, with more than half the world’s population being infected [1]. As is different from other infectious diseases, attempts to cure *H. pylori* infection with a single therapeutic attempt are unsuccessful in many patients. This largely depends on the increasing prevalence of primary antibiotic resistance [16]. Moreover, *H. pylori* eradication following therapy failures remains challenging for clinicians. Indeed, when an antibiotic was used, secondary resistance occurred in virtually all eradication failure strains, preventing its further use. This largely applies to clarithromycin, levofloxacin and metronidazole; whilst both primary and secondary resistance towards amoxicillin and tetracycline remain very low [49]. In 2017, the World Health Organization categorized clarithromycin-resistant *H. pylori* as a “high-priority” bacterium [50]. Moreover, bacterial culture availability for antibiotic susceptibility testing towards the few available antibiotics is scant, because it requires a dedicated microbiology laboratory with a specialized staff. Furthermore, *H. pylori* has slow bacterial growth and particular nutritional requirements, and culture-based methods are time-consuming (10 to 15 days), expensive, technically challenging, susceptible to inter-observer variability, and are not able to detect hetero-resistance status [11]. For these reasons, even tailored regimens were found to be no better than empiric therapies [51]. Therefore, standard therapies are empirically administered for *H. pylori* infection retreatment in clinical practice. This study evaluated the performance of different standard antibiotic regimens as rescue therapies for *H. pylori* eradication in Italy, and data from nearly five thousand patients were collected. We found that bismuth-based quadruple therapies and the sequential regimen achieved a similarly high eradication rate as second-line treatments, so that they might be administered in reversed sequence according to the initial regimen used. As far as the third-line therapy is concerned, better results were observed with levofloxacin-based and Pylera^®^, with similar eradication rates of 85–88%. Moreover, our data suggest that 14-day levofloxacin-based triple therapy could be more effective than the 10-day regimen, although the longer regimen was tested in only very few patients (21/25, 84% *vs* 1147/1452, 78.9%; *p* = 0.7). However, this result is in agreement with data reported by a systematic review of international studies, showing a better performance for the 14 days regimen [52]. Therefore, the longer 14-day schedule should be preferred when choosing the levofloxacin-based regimen for rescue therapy. As for the fourth to fifth attempt, more consistent data are available only for Pylera^®^ and rifabutin-based triple therapy, with a superiority of the quadruple regimen, even though the eradication rate was lower than 80%. Following these considerations and based on available data, an ‘empirical rescue package’ might be proposed for clinical practice, as reported in Figure 2. A limitation of this therapeutic sequence is that all regimens, apart from bismuth-based quadruple therapies, include amoxicillin, so that it is not suitable for patients with a penicillin allergy. On the other hand, a tailored sequence has been reported for these patients [53]. Of note, data from a systematic review, including recent studies on rescue therapy for *H. pylori* infection performed in European countries, found similar results [54]. Therefore, the therapeutic sequence we proposed could also be applied in other geographic areas. Another limitation of our review was that it did not evaluate the quality of the included studies, so that the strength of information was not immediately evident. Moreover, the type of first-line therapy used in different studies before the second-line therapy was not considered in our analysis. Unexpectedly, no data were available on concomitant, hybrid and high-dose dual therapy. Interest in the last regimen was recently renewed, even as a first-line therapy [54]. Data of 3 meta-analyses found that dual therapy with high-dose PPI and amoxicillin achieved similar eradication rates to levofloxacin-based, rifabutin-based and bismuth-based quadruple therapies when used as rescue therapy, but with a significantly lower side-effect rate [55,56,57]. However, only data from Asian studies were considered, and no Italian data were available. Therefore, testing for high-dose dual therapy as rescue therapy in Italy is urged in order to increase the ‘weapons in our therapeutic armamentarium’. Indeed, such a therapy might be introduced before rifabutin, aiming to limit the use to avoid bacterial resistance, when considering its usefulness for tuberculosis treatment in AIDS patients and its potential use for multidrug-resistant pathogens such as the so-called ESKAPE organisms (an acronym for *Enterococcus faecium, S. aureus, Klebsiella pneumoniae, Acinetobacter baumannii, Pseudomonas aeruginosa*, and *Enterobacter* species) [58]. In conclusion, data obtained through our literature review found that standard therapies for *H. pylori* eradication can be used when following an appropriate sequence, allowing clinicians to improve the cure rate without resorting to bacterial culture.

## 4. Materials and Methods

### 4.1. Literature Review

Separate computer-assisted searches were performed using PubMed. The search was performed on studies published in the last 15 years, from 1 January 2005 through to 31 December 2020, using the exploded medical subject heading terms “*Helicobacter pylori”* (“*Helicobacter*” OR “*H. pylori*”) AND “therapy” (OR “treatment” OR “eradication”) AND “Italy”. A manual search of references in the retrieved full papers was performed to search for missed studies. Only therapeutic studies concerning rescue (at least 1 previous failed attempt) therapies by using the most common regimens prescribed were considered (Table 2). Studies including modified drug combinations (i.e., doxycycline, ciprofloxacin, moxifloxacin, azithromycin, etc.) were not considered. Following abstract evaluation, the full text of all relevant studies was retrieved, and manual searches of reference lists from identified relevant articles were performed to identify any additional studies that might have been missed. When more than one publication from the same investigator or group was available, only the most updated version including the entire sample size was considered. Studies that included pediatric patients, case series with fewer than 5 patients, and those in a language other than English were excluded. Two investigators (V.D.F. and A.Z.) independently extracted data according to a specifically designed database, and conflicting data were resolved by a third investigator (L.G.).

### 4.2. Statistical Analysis

The eradication rates and their 95% confidence intervals at both intention-to-treat (ITT) and per-protocol (PP) analyses were computed for each subgroup. A comparison of cure rates was performed by using the Chi-squared test. Differences were considered significant at a 5% probability level. Analyses were performed by using Statsoft version 7.1 (StatSoft Europe GmbH, 22301 Hamburg, Germany) program for Windows 10.

## Figures and Tables

**Table 1:** Eradication rates at intention-to-treat (ITT) and per-protocol (PP) analysis.

Therapy Regimen	Second-Line % (95% CI)	Third-Line % (95% CI)	≥Fourth-Line % (95% CI)
Levofloxacin (N)	1273	151	63
ITT	78.7 (76.5–81)	84.7 (79–90.5)	74.6 (63.8–85.3)
PP	81.6 (79.3–84)	88.2 (83–93.5)	77 (66.5–87.6)
Pylera–quadruple (N)	873	222	164
ITT	90.6 (88.6–92.5)	88.2 (84–92.5)	77.4 (71–83.8)
PP	94 (92.2–95.7)	91.4 (86.5–96.2)	81.9 (75.1–88.7)
Bismuth–quadruple (N)	154	27	-
ITT	92.8 (88.7–96.9)	51.8 (33–70.6)	-
PP	98.6 (95.9–100)	58.3 (38.6–78)	-
Rifabutin (N)	1009	428	301
ITT	65.9 (62.9–68.8)	77.3 (73.3–81.3)	66.4 (61.1–71.7)
PP	76.4 (73.6–79.2)	82.1 (78.3–85.8)	73.3 (67.8–78.2)
Sequential (N)	118	29	18
ITT	89.8 (84.3–95.2)	79.3 (64.5–94)	77.2 (51.5–92.9)
PP	95.5 (91.6–99.3)	85.1 (71.7–98.5)	81.2 (62.1–100)

ITT: intention-to-treat; PP: per-protocol analysis; CI: confidence interval.

**Table 2:** Therapy regimens for *Helicobacter pylori* eradication.

Therapy	Schedule	Duration
Sequential	Esomeprazole 40 mg and amoxycillin 1 g, all b.i.d for 5 days followed by: Esomeprazole 40 mg, clarithromycin 500 mg, and tinidazole 500 mg, all b.i.d for 5 days	10 days
Pylera^®^	3 tablets q.i.d. and omeprazole 20 mg b.i.d	10 days
Levofloxacin	Esomeprazole 40 mg, levofloxacin 250 mg, and amoxycillin 1 g, all b.i.d	14 days
High-dose dual	Esomeprazole 40 mg and amoxycillin 1 g, all t.i.d	14 days
Rifabutin	Esomeprazole 40 mg and amoxycillin 1 g b.i.d plus rifabutin 150 mg q.d	12 days

b.i.d: twice a day; q.i.d: four times a day; t.i.d: three times a day; q.d: once a day.

## Data Availability

Data available in a publicly accessible repository. The data presented in this study are openly available in the PubMed database.
